# Hippuric Acid Promotes Renal Fibrosis by Disrupting Redox Homeostasis via Facilitation of NRF2–KEAP1–CUL3 Interactions in Chronic Kidney Disease

**DOI:** 10.3390/antiox9090783

**Published:** 2020-08-25

**Authors:** Bowen Sun, Xifan Wang, Xiaoxue Liu, Longjiao Wang, Fazheng Ren, Xiaoyu Wang, Xiaojing Leng

**Affiliations:** 1 Key Laboratory of Precision Nutrition and Food Quality, Key Laboratory of Functional Dairy, Ministry of Education, College of Food Science & Nutritional Engineering, China Agricultural University, Beijing 100083, China; sunbwfood@163.com (B.S.); wangxfan@126.com (X.W.); lxiaoxue0828@163.com (X.L.); wanglongjiao95@163.com (L.W.); renfazheng@cau.edu.cn (F.R.); 2 Beijing Advanced Innovation Center for Food Nutrition and Human Health, College of Food Science & Nutritional Engineering, China Agricultural University, Beijing 100083, China

**Keywords:** hippuric acid, protein-bound uremic toxin, renal fibrosis, oxidative stress, antioxidation, NRF2, NRF2 ubiquitination

## Abstract

Chronic kidney disease (CKD) is characterized by the accumulation of protein-bound uremic toxins (PBUTs), which play a pathophysiological role in renal fibrosis (a common pathological process resulting in CKD progression). Accumulation of the PBUT hippuric acid (HA) is positively correlated with disease progression in CKD patients, suggesting that HA may promote renal fibrosis. Oxidative stress is the most important factor affecting PBUTs nephrotoxicity. Herein, we assessed the ability of HA to promote kidney fibrosis by disrupting redox homeostasis. In HK-2 cells, HA increased fibrosis-related gene expression, extracellular matrix imbalance, and oxidative stress. Additionally, reactive oxygen species (ROS)-mediated TGFβ/SMAD signaling contributed to HA-induced fibrotic responses. HA disrupted antioxidant networks by decreasing the levels of nuclear factor erythroid 2-related factor 2 (NRF2), leading to ROS accumulation and fibrotic responses, as evidenced by NRF2 activation and knockdown. Moreover, NRF2 levels were reduced by NRF2 ubiquitination, which was regulated via increased interactions of Kelch-like ECH-associated protein 1 with Cullin 3 and NRF2. Finally, renal fibrosis and redox imbalance promoted by HA were confirmed in rats. Importantly, sulforaphane (NRF2 activator) reversed HA-promoted renal fibrosis. Thus, HA promotes renal fibrosis in CKD by disrupting NRF2-driven antioxidant system, indicating that NRF2 is a potential therapeutic target for CKD.

## 1. Introduction

Approximately 10–15% of the world population suffers from chronic kidney disease (CKD), making it a serious public health and social issue [1]. CKD is a progressive disease in which sustained damage to the kidney structure ultimately results in irreversible kidney failure, defined as the end-stage renal disease (ESRD) [2]. Renal fibrosis, a common pathological process of CKD, results in the multiple types of CKD progress to ESRD [3,4], in which original functional tissue is replaced by connective tissue, resulting in loss of the kidney architecture [5]. During CKD, renal fibrosis progression is closely related to the accumulation of uremic toxins, which is caused by renal dysfunction in CKD [6]. Accumulation of these toxins exerts a destructive influence on kidney tissue and cells, thereby promoting the progression of renal fibrosis and aggravating the deterioration of renal function, eventually forming a vicious circle [7]. Removal of uremic toxins by peritoneal dialysis, oral charcoal adsorbents, or high-flux biocompatible hemodialysis suspends the development of CKD and deterioration of renal function according to both clinical and experimental studies [8,9,10].

Protein-bound uremic toxins (PBUTs) are the most important uremic toxins, as they are not dialyzable and are difficult to remove [7]. Recently, the relationship between PBUTs and renal fibrosis has been the subject of great interest and active research. Published studies on PBUTs suggest that indoxyl sulfate (IS) and *p*-cresol sulfate (PCS) promote renal fibrosis in 5/6th nephrectomy (5/6NX) rats and renal tubular HK-2 cells [7]. However, PBUTs need to be further explored and investigated. The PBUT hippuric acid (HA) is generated from dietary polyphenols by the gut microbiota [11] and its serum concentration is significantly higher than those of IS and PCS in patients with CKD according to data from the European Uremic Toxin Work Group (EUTox). Recently, clinical and metabolomic studies revealed that increased serum HA levels are positively correlated with the risk of kidney failure and dysfunction [12,13,14]. Furthermore, a study reported that HA accelerates kidney damage in 5/6NX rats based on histopathological analysis [15]. These findings suggest that HA may be a novel uremic toxin that promotes the progression of renal fibrosis in CKD. However, the underlying mechanisms of HA remain largely unknown.

The pathogenic mechanisms of some PBUTs, such as IS and PCS, have been determined. IS and PCS initially appear to damage critical functional units in the kidney, known as renal tubules, leading to their pathological changes, such as fibrogenic cytokine formation and fibrotic gene expression, thereby promoting the development of renal fibrosis in CKD [16,17,18]. The critical mechanism of PBUT-induced tubular damage is considered to involve the activation of oxidative stress, inflammation, and the renin-angiotensin system, among which oxidative stress is the most important driving force of tubular damage and a typical feature of renal fibrosis [18,19]. Interestingly, HA also exhibits oxidative stress-associated toxicity. HA is involved in forming reactive oxygen species (ROS) in endothelial cells [20] and promoted endothelial dysfunction by increasing mitochondrial ROS in a 5/6NX rat model [21]. These findings suggest that the nephrotoxicity of HA is potentially related to oxidative stress.

Oxidative stress results from the disruption of redox homeostasis (i.e., the imbalance between oxidative and antioxidative reactions), which manifests as ROS accumulation. Most studies of uremic toxin-caused oxidative injuries have focused on the generation of oxygen free radicals [17,22,23]. However, antioxidative defenses also have an important role in the onset and development of many diseases wherein redox imbalances occur, including renal fibrosis, acute kidney injury, and lupus-like autoimmune nephritis [24]. The antioxidant defense network is regulated by nuclear factor erythroid 2-related factor 2 (NRF2), which activates numerous antioxidants and phase II detoxifying enzymes to eliminate ROS accumulation and maintain redox homeostasis [25]. NRF2 activity depends on the regulation of protein turnover by the ubiquitin-proteasome degradation system, which is dominated by E3 ubiquitin ligase complex, a NRF2–Kelch-like ECH associated protein 1 (KEAP1)–Cullin 3 (CUL3) complex [26,27]. Once NRF2 is ubiquitinated, it is recognized and degraded by 26S proteasomes in the cytoplasm, preventing its translocation into the nucleus [28]. In turn, under oxidative and electrophilic stimulation, NRF2–KEAP1–CUL3 interactions are disrupted, and NRF2 is dissociated from the complex and translocated to the nucleus, activating downstream antioxidant genes [29]. At present, the association between PBUT-promoted renal fibrosis and the NRF2-driven antioxidant system remains unclear.

This study was conducted to assess the ability of HA to promote kidney fibrosis by disrupting redox homeostasis in vitro and in vivo. Furthermore, we investigated the mechanism underlying HA impairment of the NRF2-driven antioxidant system. These findings improve the understanding of the pathogenesis of PUBT-promoted renal fibrosis in CKD. Additionally, this work provides a foundation for improved therapies for patients with CKD by targeting NRF2.

## 2. Materials and Methods 

### 2.1. Materials, Reagents, and Antibodies 

HA, *N*-acetylcysteine (NAC), diphenylene iodonium (DPI), and dimethyl sulfoxide (DMSO) were provided by Sigma Aldrich company (St. Louis, MO, USA) and MG132 was obtained in Aladdin (Shanghai, China). Sulforaphane (SFN) was from TRC company (North York, ON, Canada). Antibodies against phosphorylated SMAD2 (p-SMAD2), p-SMAD3, SMAD2, SMAD3, SMAD4, CUL3, tissue inhibitor of metalloproteinases-1 (TIMP1), and ubiquitin (Ub) were obtained by CST company (Danvers, MA, USA). Antibodies containing collagen-I (COL1A1), E-cadherin (CDH1), alpha-smooth muscle actin (ACTA2), vimentin (VIM), heme oxygenase-1 (HO1), KEAP1, SNAI1, matrix metalloproteinase-9 (MMP9), NAD(P)H quinone dehydrogenase (NQO1), β-ACTIN, histone H3, and secondary antibodies were purchased from Proteintech (Chicago, IL, USA). Antibodies against NADPH oxidase 4 (NOX4) and NRF2 were provided by Abcam company (Cambridge, UK).

### 2.2. Cell Culture

HK-2 cells (human renal tubular epithelial cells, CRL-2190™), a recognized cell line for studying the nephrotoxicity of PUBTs [7], were supplied by ATCC (Manassas, VA, USA) and cultured in the recommended keratinocyte serum-free medium (Gibco, Grand Island, NY, USA) in 95% air and 5% CO_2_ at 37 °C. Then the cells were treated with HA at concentrations of 0, 62.5, 125, 250, 500, and 1000 µM to assess the concentration–response relationship, when these cells were grown to 60–70% confluence. In other experiments, HK-2 cells were pretreated with NAC (1000 µM, 1 h), DPI (50 µM, 1 h), SFN (0–20 µM, 24 h), or MG132 (10 µM, 1 h), followed by treatment with 1000 µM HA. DMSO (0.1%) was used as vehicle control.

### 2.3. Animal Experiments

All procedures and protocols involving rats were approved by the Ethical Committee of Experimental Animal Care at China Agricultural University and complied with ARRIVE guidelines (ethical code: KY19034). Male Sprague Dawley rats at the age of 7 weeks were housed in a pathogen-free environment. The environment was that with a humidity of 50–70%, temperature of 22–24 °C, and the day and night cycle was 12/12 h. These rats were given a normal laboratory diet (Shanghai Puluteng Biological Technology Co., Ltd., Shanghai, China) with ad libitum access to water. After 1 week of adaptation, fifty rats were subjected to either sham surgery (kidney exposure operation only) or 5/6NX surgery [30]. The 5/6NX model commonly used to simulate typical CKD symptoms and study PUBT-promoted renal fibrosis, such as IS and PCS [7,15,17,18]. Two weeks after surgery (experimental week 1), excluding the rats that died during the operation, rats in the sham group (*n* = 9) were treated with vehicle (0.5% DMSO), and 5/6NX rats were randomized into four groups (*n* = 9 each) and treated as follows: (1) a 5/6NX group, 0.5% DMSO; (2) a 5/6NX + SFN group, 2.5 mg/kg SFN; (3) a 5/6NX + HA group, 100 mg/kg HA; (4) a 5/6NX + HA + SFN group, 100 mg/kg HA and 2.5 mg/kg SFN. All treatments were administered intraperitoneally, five times per week for 10 weeks. 

### 2.4. Cell Viability 

The viability of HK-2 cells was determined using Cell Counting Kit-8 (Beyotime, Shanghai, China) at 450 nm on a microplate reader (Bio-Rad, Hercules, CA, USA), according to the manufacturer’s recommendations. The baseline level was measured on a microplate containing the cell culture medium, DMSO (0.1%), and CCK-8 solution but no cells (*n* = 6).

### 2.5. Quantitative RT-PCR

Total RNA of HK-2 was obtained by TRIzol solution (Invitrogen, Carlsbad, CA, USA). Reverse transcription was carried out by 5× All-In-One RT MasterMix (Applied Biological Materials, Inc., Richmond, BC, Canada). The quantitative polymerase chain reaction was performed on a 7900HT instrument (Applied Biosystems, Foster City, CA, USA) by TB Green Premix Ex Taq (Takara Biomedical Technology Co., Ltd., Shiga, Japan) following the manufacturers’ protocol. The sequences of primers were shown in Supplementary Materials (Table S1).

### 2.6. Western Blotting (WB)

Total proteins of cells were extracted by the ice-cold RIPA solution (Beyotime). Nuclear and Cytoplasmic Protein Extraction kit (Beyotime) was applied to obtain the nuclear and cytoplasmic proteins. Equal-concentration protein samples were separated using SDS-PAGE. Then the samples were shifted to polyvinylidene difluoride membranes (EMD Millipore, Billerica, MA, USA). The membranes were blocked at 4 °C overnight and then were incubated using the indicated primary antibody. Next, the membranes were incubated with a secondary antibody. The enhanced chemiluminescence (EMD Millipore) was used to develop the signals.

### 2.7. Immunofluorescence Assays

HK-2 cells grown in 6 well plates were fixed for about 10 min using paraformaldehyde (4%) and then permeabilized about 10 min using Triton X-100 (0.25%) at 25 °C. Then, the samples were blocked for 1 h using blocking buffer (Beyotime). After washing, the samples were incubated with primary antibodies and then secondary fluorescent antibodies. The nuclei of cells were stained by DAPI solution (Solarbio, Beijing, China) for 5 min.

### 2.8. ROS and Hydrogen Peroxide (H_2_O_2_) Assays

Total intracellular ROS and H_2_O_2_ concentrations were determined using commercial ROS and H_2_O_2_ assay kits (Beyotime) in accordance with the manufacturer’s instruction manuals.

### 2.9. NRF2 Knockdown

The cells were transfected with a *NRF2*-specific small interfering RNA (siRNA) or with a siRNA control (Table S2) by Lipofectamine 2000 (Thermo Fisher Scientific, Waltham, MA, USA) in accordance with the manufacturer’s instruction manuals.

### 2.10. Coimmunoprecipitation (Co-IP)

NRF2 ubiquitination and protein–protein interactions were determined by Co-IP using a kit of Pierce™ Crosslink IP (Thermo Fisher Scientific) following the manufacturers’ protocol. Co-IP products were boiled in loading buffer and subjected to WB with the appropriate antibodies.

### 2.11. Molecular Docking

The HA structure was obtained from the PubChem database (CID 464). The KEAP1 structure (PDB 4N1B) was obtained by the Protein Data Bank. Water molecules, acetate ion, and the original ligand molecules 2FS were deleted. Subsequently, HA was docked into KEAP1 active sites using Autodock Vina software 1.1.2 (Scripps Research Institute, La Jolla, CA, USA). The interaction model of minimum binding energy was selected.

### 2.12. Serum Biochemical Examination

Rats serum creatinine (SCr) and blood urea nitrogen (BUN) contents were evaluated using a BS-420 automated biochemical analyzer (Mindray, Shenzhen, Guangdong, China). 

### 2.13. HA Measurement

HA in rat serum was measured by ultra-performance liquid chromatography-triple quadruple-mass spectrometry (Bruker, Bremen, Germany). HA was separated on an InertSustain AQ-C18 column (inner diameter 1.9 µm, 2.1 × 150 mm, Shimadzu, Kyoto, Japan). The volume of injection was 10 µL, the temperature of the column was 40 °C, and the rate of flow was 0.25 mL/min. The mobile phase contained (A) ultrapure water and (B) acetonitrile (both containing 0.1% formic acid). The gradients of elution were 0–2 min, 0% B; 2–5 min, 0–15% B; 5–11 min, 15% B; 11–15 min, 15–55% B; 15–16 min, 55–95% B; and 16–20 min, 95–0% B. Mass spectrometry was performed in multiple reaction-monitoring modes with negative ionization. Electrospray settings of 4000 V positive and 3500 V negative spray voltage, 400 °C needle temperature, and 350 °C cone temperature were used. The aux gas was high-purity nitrogen. The quantitation of HA in rat serum was performed by constructing calibration curves using the standard HA (TRC, Inc., San Diego, CA, USA).

### 2.14. Histological and Immunohistochemical Analyses

Kidney samples were fixed for 24 h using paraformaldehyde (4%) and were then embedded using paraffin. Thin sections were deparaffinized, rehydrated, and stained by corresponding dyes (Masson’s trichrome and periodic acid–Schiff (PAS)). Fibrotic areas and sclerosis indexes were quantified as previously described [31]. For immunohistochemistry, the samples were stained with the specified antibodies (anti-COL1A1, 1:1000; anti-VIM, 1:1500; anti-ACTA2, 1:1500; and anti-NRF2, 1:500). All samples (*n* = 9 per group) were analyzed and calculated by IPP software 6.0 (Media Cybernetics, Inc., Rockville, MD, USA).

### 2.15. Malondialdehyde and Antioxidant Enzyme Assays

Malondialdehyde (MDA) in kidney tissues was assessed with an MDA assay kit (Beyotime) following the manufacturers’ protocol. The activity of superoxide dismutase (SOD), catalase (CAT), and glutathione peroxidase (GSH-Px) in renal samples were quantified with enzyme-specific assay kits (Beyotime) following the manufacturers’ protocol.

### 2.16. Statistical Analyses 

**Figure 1:**
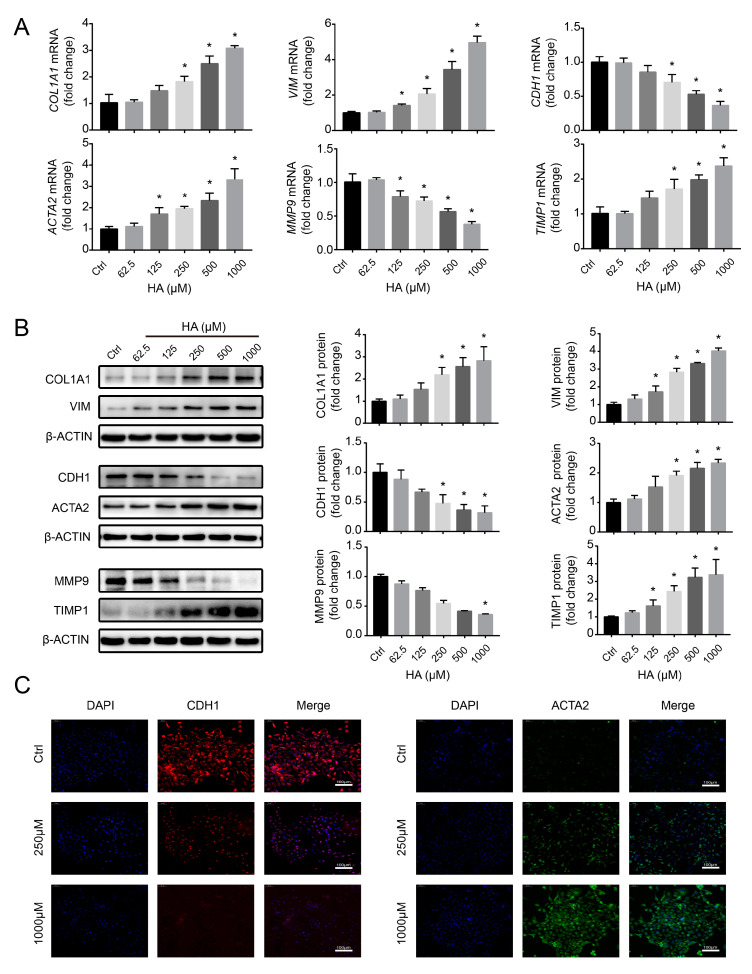
Effects of hippuric acid (HA) on fibrotic gene expression in HK-2 cells. (**A**) Relative mRNA and (**B**) protein expression levels of collagen-I (COL1A1), vimentin (VIM), E-cadherin (CDH1), alpha-smooth muscle actin (ACTA2), matrix metalloproteinase-9 (MMP9), and tissue inhibitor of metalloproteinases-1 (TIMP1) after treatment with 0–1000 μM HA. Quantification was performed by densitometry. (**C**) Immunofluorescence staining for CDH1 (red) and ACTA2 (green) after treatment with 250 and 1000 μM HA. Nuclei (blue) were stained with DAPI. Bar = 100 μm. Data are presented as the mean ± standard deviation. * *p* < 0.05 vs. control (Ctrl) cells. Experiments were repeated three to four times.

All values were expressed as mean ± standard deviation and analyzed using SPSS 21.0 (SPSS, Inc., Chicago, IL, USA). The Gaussian distribution of data was analyzed by the Shapiro–Wilk test. Statistical comparison was measured by one-way ANOVA followed by Tukey’s post-test (data with Gaussian distribution) or Kruskal–Wallis followed by Dunn’s post-test (data without Gaussian distribution). The data that does not conform to the normal distribution are the WB results of MMP9 (Figure 1B), NOX4 (Figure 3B), HO1 (Figure 4B), NQO1 (Figure 4B), VIM (Figure 5B), ACTA2 (Figure 5B) and CUL3 (Figure 6C). Differences were considered significant when *p* < 0.05.

## 3. Results

### 3.1. HA Displays Strong Potential to Cause Fibrotic Responses In Vitro

To evaluate the potential role of HA in promoting fibrotic responses, HK-2 cells were incubated with HA for 24 h at concentrations of 0–1000 µM, based on mean serum HA concentration in patients on hemodialysis (610.78 µM; EUTox data). There were no significant differences in cell viability at 0–1000 µM HA (Figure S1). Next, renal fibrosis markers were assessed, including COL1A1, VIM, CDH1, and ACTA2. HA significantly increased COL1A1, VIM, and ACTA2 mRNA and protein levels, and reduced those of CDH1 in a concentration-dependent manner (Figure 1A,B). Immunofluorescence staining for CDH1 and ACTA2 revealed similar results, with the CDH1 intensity significantly decreased and that of ACTA2 increased at 250 and 1000 µM HA compared to the control (Figure 1C). Furthermore, MMP9 and TIMP1 expression levels were assessed. HA significantly attenuated MMP9 mRNA and protein levels, whereas it significantly increased those of TIMP1 (Figure 1A,B), suggesting that HA induced extracellular matrix (ECM) imbalance. These results indicate that HA caused fibrogenic responses in HK-2 cells.

### 3.2. TGFβ/SMAD Signaling Is Involved in HA-Induced Fibrotic Responses In Vitro

**Figure 2:**
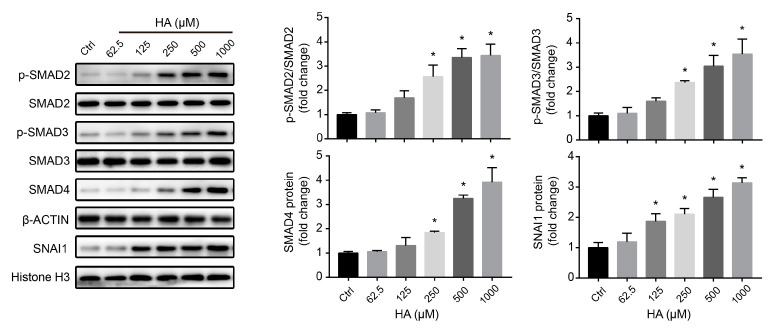
Effects of HA on TGFβ/SMAD signaling in HK-2 cells. Expression levels of key proteins in the TGFβ/SMAD signaling pathway after treatment with 0–1000 μM HA. Quantification (right) was performed by densitometry. Data are presented as the mean ± standard deviation. * *p* < 0.05 vs. control (Ctrl) cells. Experiments were repeated three times.

To elucidate the underlying molecular mechanism of the effects of HA on fibrotic responses, TGFβ/SMAD signaling was investigated in HK-2 cells. Phosphorylation of SMAD2 and SMAD3, as well as the protein expression levels of SMAD4, significantly increased by HA treatment in a concentration-dependent manner (Figure 2). Moreover, expression of the transcription factor SNAI1 was significantly enhanced in renal tubular cells (Figure 2). These data suggest that HA induced fibrotic responses by activating TGFβ/SMAD signaling.

### 3.3. HA Induces Fibrotic Responses via ROS-Activated TGFβ/SMAD Signaling In Vitro

**Figure 3:**
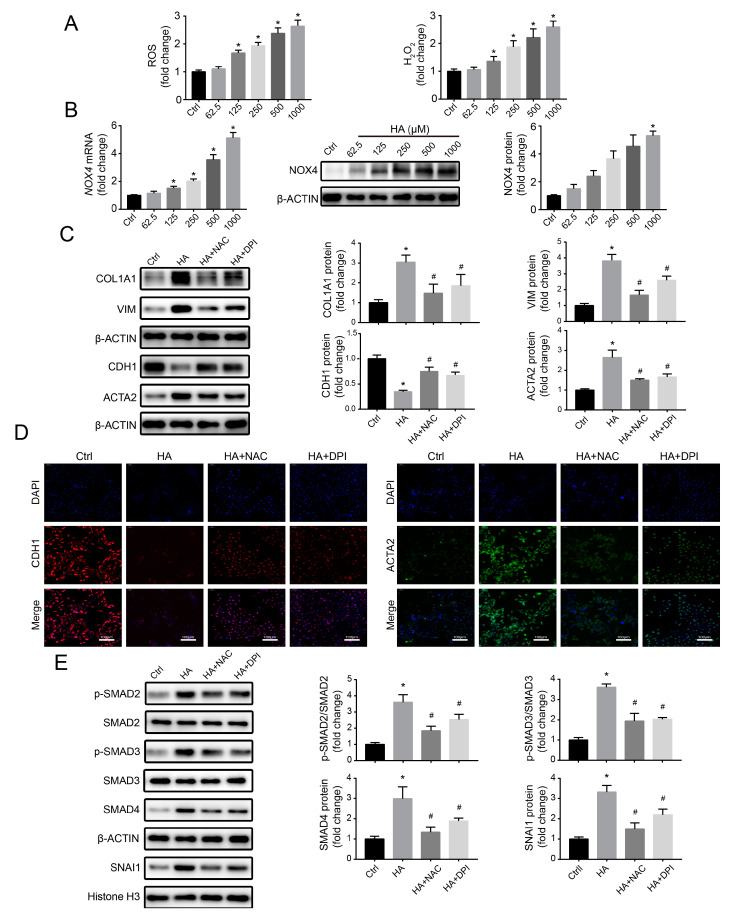
Role of oxidative stress in hippuric acid (HA)-induced fibrotic gene expression in HK-2 cells. (**A**) Levels of reactive oxygen species (ROS) and hydrogen peroxide (H_2_O_2_) after treatment with 0–1000 μM HA. (**B**) Relative mRNA and protein expression levels of NADPH oxidase 4 (NOX4) after treatment with 0–1000 μM HA. (**C**) Levels of protein expression of collagen-I (COL1A1), vimentin (VIM), E-cadherin (CDH1), and alpha-smooth muscle actin (ACTA2) in cells pretreated with 1000 µM *N*-acetylcysteine (NAC), 50 µM diphenylene iodonium (DPI), or the vehicle, followed by treatment with 1000 µM HA. (**D**) Immunofluorescence staining for CDH1 (red) and ACTA2 (green) after pretreatment with 1000 µM NAC, 50 µM DPI, or the vehicle, followed by treatment with 1000 µM HA. Nuclei (blue) were stained with DAPI. Bar = 100 μm. (**E**) Expression levels of key proteins in the TGFβ/SMAD signaling pathway in cells pretreated with 1000 µM NAC, 50 µM DPI, or the vehicle, followed by treatment with 1000 µM HA. Quantification was performed by densitometry. Data are presented as the mean ± standard deviation. * *p* < 0.05 vs. control (Ctrl) cells; # *p* < 0.05 vs. cells treated with HA only. Experiments were repeated three to five times.

To investigate whether oxidative stress is involved in HA-induced fibrotic responses, the levels of oxygen free radicals were analyzed in HK-2 cells. Increasing HA concentrations not only significantly augmented the production of ROS and H_2_O_2_ but also promoted the mRNA and protein expression of NOX4, a known enzymatic source of ROS in the renal system (Figure 3A,B). These data indicate that HA induced oxidative stress in tubular cells.

Further, HK-2 cells were pretreated with the free radical scavenger NAC (1000 µM) and NOX4 inhibitor DPI (50 µM), followed by incubation with HA (1000 µM). NAC and DPI suppressed ROS and H_2_O_2_ production in HK-2 cells treated with 1000 µM HA (Figure S2). Moreover, significant decreases in COL1A1, VIM, and ACTA2 protein levels and an increase in the CDH1 level were observed in both the HA + NAC and HA + DPI groups, compared with those in the HA-only group (Figure 3C). Consistent with the WB results, immunofluorescence revealed that the two antioxidants (NAC and DPI) significantly reversed the reduction in CDH1 expression and enhancement of ACTA2 expression (Figure 3D). Additionally, HA-induced elevation in the levels of key proteins (p-SMAD2, p-SMAD3, SMAD4, and SNAI1) in the TGFβ/SMAD signaling pathway was reversed by NAC and DPI pretreatment of HK-2 cells (Figure 3E). Together, these data indicated that the HA-induced fibrotic responses were mediated by ROS activation of the TGFβ/SMAD pathway in renal tubular cells.

### 3.4. Effects of HA Treatment on the NRF2-Driven Antioxidant Pathway In Vitro

**Figure 4:**
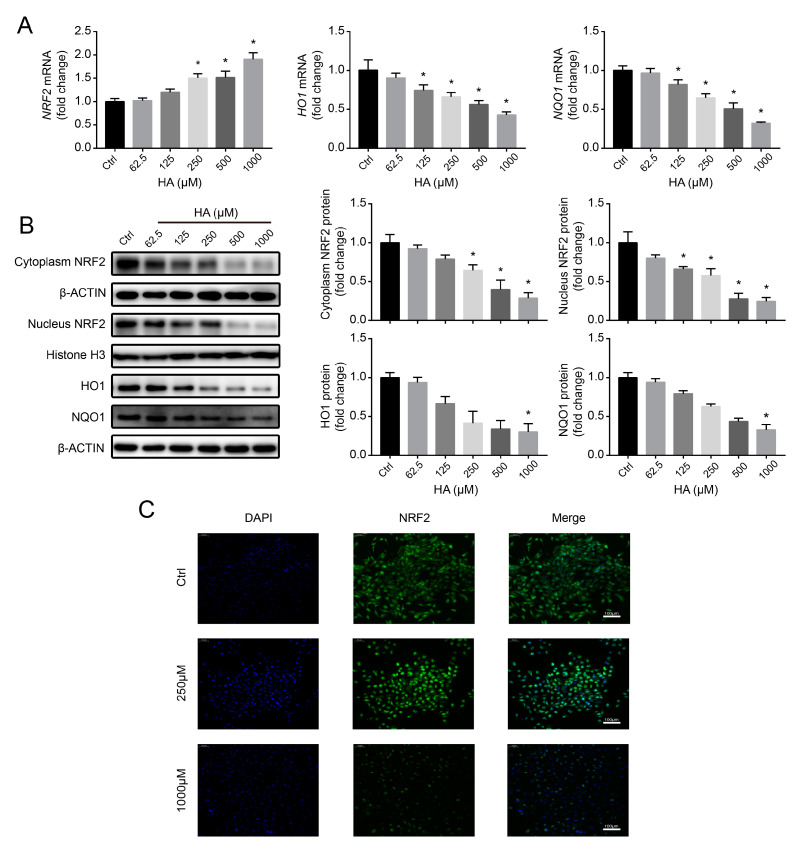
Effects of HA on the nuclear factor erythroid 2-related factor 2 (NRF2)-mediated antioxidant system in HK-2 cells. (**A**) Levels of mRNA expression of *NRF2*, heme oxygenase-1 (*HO1*), and NAD(P)H quinone dehydrogenase (*NQO1*) after treatment with 0–1000 μM HA. (**B**) Levels of protein expression of cytoplasmic and nuclear NRF2, HO1, and NQO1 after treatment with 0–1000 μM HA. Quantification was performed by densitometry. (**C**) Immunofluorescence staining for NRF2 (green) after treatment with 250 and 1000 μM HA. Nuclei (blue) were stained with DAPI. Bar = 100 μm. Data are presented as the mean ± standard deviation. * *p* < 0.05 vs. control (Ctrl) cells. Experiments were repeated three to four times.

Under ROS stress, the NRF2-mediated antioxidant system can be activated and eliminate ROS, thereby preventing the initiation of oxidative stress [32,33]. To assess the effect of HA on the antioxidant network, NRF2 and its downstream antioxidant enzymes (HO1 and NQO1) levels were measured. The expressions of mRNA and protein of HO1 and NQO1 were notably reduced with increasing concentrations of HA (Figure 4A,B), suggesting that the NRF2-driven antioxidant pathway was disrupted. Interestingly, HA induced a concentration-dependent increase in *NRF2* mRNA levels, accompanied by a significant decrease in cytoplasmic and nuclear NRF2 protein expression (Figure 4A,B). Consistent with the WB data, NRF2 immunofluorescence significantly decreased at 250 and 1000 µM HA compared to the control (Figure 4C). These results suggested that HA inhibited the antioxidant pathway by downregulating NRF2 protein.

### 3.5. NRF2-Driven Antioxidant Network Mediates HA-Induced Fibrotic Responses In Vitro

**Figure 5:**
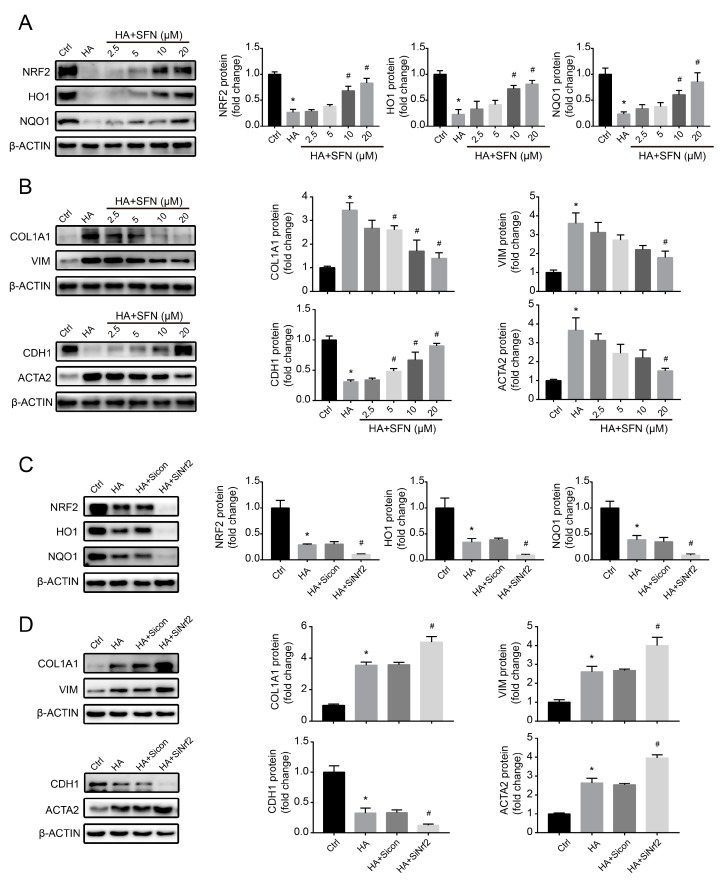
Role of nuclear factor erythroid 2-related factor 2 (NRF2) in hippuric acid (HA)-induced fibrotic gene expression in HK-2 cells. (**A**) Levels of protein expression of NRF2, heme oxygenase-1 (HO1), and NAD(P)H quinone dehydrogenase (NQO1) in cells pretreated with 0–20 µM sulforaphane (SFN), followed by treatment with 1000 µM HA. (**B**) Levels of protein expression of collagen-I (COL1A1), vimentin (VIM), E-cadherin (CDH1), and alpha-smooth muscle actin (ACTA2) in cells pretreated with 0–20 µM SFN, followed by treatment with 1000 µM HA. (**C**) Levels of protein expression of NRF2, HO1, and NQO1 after cell transfection with the control or *NRF2*-specific small interfering RNA (siRNA), followed by treatment with 1000 µM HA. (**D**) Levels of protein expression of COL1A1, VIM, CDH1, and ACTA2 after cell transfection with the control or *NRF2*-specific siRNA, followed by treatment with 1000 µM HA. Quantification was performed by densitometry. Data are presented as the mean ± standard deviation. * *p* < 0.05 vs. control (Ctrl) cells; # *p* < 0.05 vs. cells treated with HA only or HA + control siRNA. Experiments were repeated three times.

To investigate the potential effect of NRF2 on fibrotic toxicity of HK-2 cells caused by HA, we used the NRF2 activator SFN (0–20 μM) to reverse HA-induced NRF2 protein downregulation and then measured the changes in the expression of key genes related to renal fibrosis. Compared with that in HA-only treatment, ROS and H_2_O_2_ production significantly suppressed following SFN + HA treatment in a concentration-dependent way, and NRF2, HO1, and NQO1 levels significantly upregulated (Figure S3 and Figure 5A), suggesting that HA-induced ROS accumulation and dysfunction of the antioxidant network were suppressed by SFN. Furthermore, NRF2 activation significantly reversed HA-induced elevation of COL1A1, VIM, and ACTA2 levels and reduction of CDH1 levels (Figure 5B), suggesting that HA-induced fibrotic responses were alleviated by NRF2 activation.

To better clarify the role of NRF2 in HA-induced fibrotic responses, an NRF2-specific siRNA was introduced into HK-2 cells. NRF2 silencing exacerbated HA-induced ROS and H_2_O_2_ generation and downregulated the NRF2-dependent antioxidant pathway (Figure S4 and Figure 5C). Moreover, HA-induced upregulation of COL1A1, VIM, and ACTA2 levels, and downregulation of CDH1 was further augmented by NRF2 knockdown (Figure 5D). In these experiments, the siRNA control group was not significantly different from the HA-only control (Figure 5C,D), displaying no additional siRNA effects on HK-2 cells. Overall, these data indicate that the NRF2 antioxidant defense system, which was disrupted by downregulation of NRF2 protein, played a vital role in HA-induced fibrotic responses.

### 3.6. HA Increases NRF2 Ubiquitination by Increasing E3 Ubiquitin Ligase Activity In Vitro

**Figure 6:**
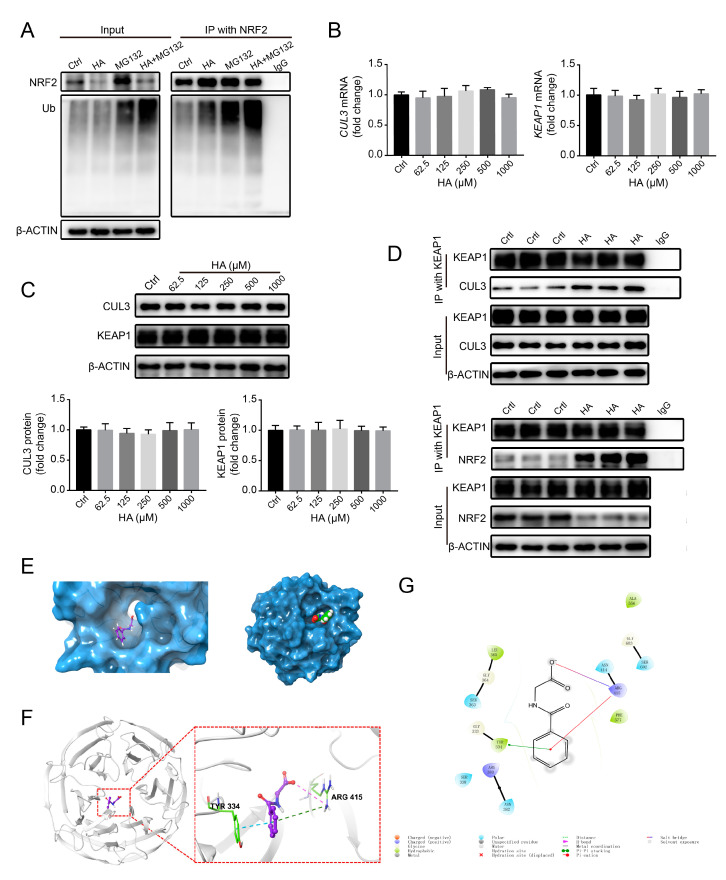
Effects of HA on NRF2 ubiquitination and E3 ubiquitin ligase in HK-2 cells. (**A**) Levels of NRF2 ubiquitination in cells pretreated with 10 μM MG132 (a proteasome inhibitor) or the vehicle, followed by treatment with 1000 μM HA. (**B**) Relative mRNA and (**C**) protein expression levels of Cullin 3 (CUL3) and Kelch-like ECH-associated protein 1 (KEAP1) after treatment with 0–1000 μM HA. Quantification was performed by densitometry. (**D**) Coimmunoprecipitation analysis of interactions between KEAP1 and CUL3 or NRF2 in cells treated with 1000 μM HA or the vehicle. (**E**) Surface representation of KEAP1 with a bound HA molecule (shown in a ball and stick format). (**F**) HA-binding site of KEAP1, shown with surface rendering. HA is depicted in a ball and stick format. (**G**) 2D detailed schematic of interactions between HA and KEAP1. Quantitative data are presented as the mean ± standard deviation. Experiments were repeated three to four times.

To explore the mechanism of the HA-induced reduction of NRF2 protein, we first determined whether HA causes NRF2 ubiquitination in HK-2 cells. The levels of ubiquitinated NRF2 significantly increased after treatment with 1000 µM HA, and similar results were obtained in the presence of 10 µM MG132 (Figure 6A), suggesting that HA promoted the ubiquitination of NRF2.

Further, to investigate whether HA affects E3 ubiquitin ligase activity, KEAP1 and CUL3 expression levels were analyzed in HK-2 cells. Interestingly, no significant changes in the mRNA and protein expression of the KEAP1 and CUL3 subunits were observed following HA treatment (Figure 6B,C). It has been reported that E3 ubiquitin ligase activity also is regulated by protein–protein interactions [34]. Therefore, Co-IP was performed to reveal such interactions. At 1000 µM HA, immunoprecipitated KEAP1 pulled down more NRF2 compared to control-treated cells, and similar results were obtained for immunoprecipitated KEAP1 and CUL3 (Figure 6D), suggesting that the interaction between KEAP1 and NRF2 or CUL3 had been strengthened. These results indicate that HA enhanced the activity of E3 ubiquitin ligase by strengthening the NRF2–KEAP1–CUL3 interactions.

To identify the HA target responsible for HA-induced NRF2 ubiquitination, a docking experiment was performed to model potential interactions between HA and KEAP1, a core component of the E3 ligase complex. HA appeared to dock into the cavity of human KEAP1 protein (Figure 6E). The benzene ring of HA formed π–π interactions with the benzene ring of Tyr334 of KEAP1 and π–cation interactions with –NH2+ of Arg415 of KEAP1, whereas the terminal carboxyl oxygen of HA formed a salt bridge with –NH2+ of Arg415 of KEAP1 (Figure 6F,G). These results suggest that HA stably binds to KEAP1 to exert its biological functions.

Collectively, these data suggest that HA directly binds to KEAP1, thereby enhancing the interactions of KEAP1 with CUL3 and NRF2, increasing the activity of E3 ligase complex, and ultimately increasing NRF2 ubiquitination and degradation.

### 3.7. HA Promotes Renal Dysfunction and Fibrosis by Disrupting the Antioxidation Function of Nrf2 In Vivo

To further explore the ability of HA to promote renal fibrosis in CKD in vivo, we generated a 5/6NX rat model to mimic typical CKD symptoms, which can simulate the actual pathogenic environment of HA-promoted renal fibrosis in patients with CKD more accurately. Compared with sham rats, 5/6NX rats showed significantly increased levels of SCr, BUN, and HA, as well as increased fibrosis area, glomerular sclerosis index, and fibrotic marker levels (COL1A1, VIM, and ACTA2), suggesting that the 5/6NX rat model was effective.

**Figure 7:**
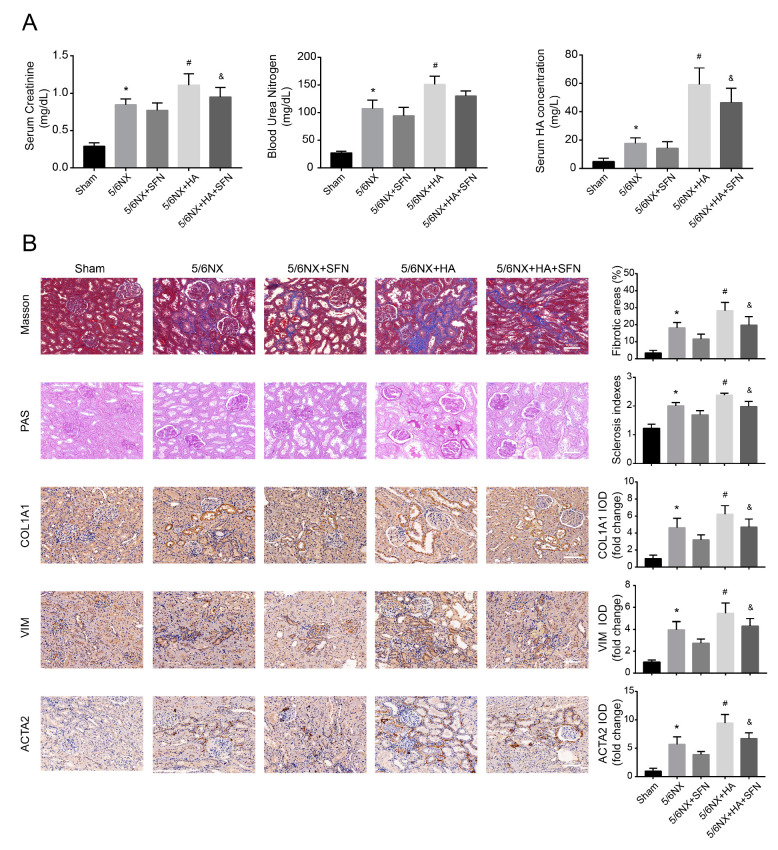
Effects of HA on dysfunction and fibrosis in rat kidneys. (**A**) Serum creatinine (SCr), blood urea nitrogen (BUN), and serum HA concentrations in rats. (**B**) Fibrotic areas, sclerosis indexes, and immunohistochemistry data for collagen-I (COL1A1), vimentin (VIM), and alpha-smooth muscle actin (ACTA2) in kidney samples. Positive areas were quantified (right) by densitometry. Bar = 100 μm. Data are presented as the means ± standard deviation (*n* = 9 per group). * *p* < 0.05 vs. sham group; # *p* < 0.05 vs. 5/6th nephrectomy (5/6NX) group; and & *p* < 0.05 vs. 5/6NX + HA group.

Serum HA levels in 5/6NX + HA rats were significantly higher than those in the 5/6NX rats (Figure 7A), suggesting that HA effectively accumulated in vivo. Compared to the 5/6NX rats, SCr and BUN contents increased in the 5/6NX + HA group (Figure 7A), suggesting that HA accumulation worsened renal function. Moreover, SFN treatment (5/6NX + HA + SFN group) attenuated the increases in SCr, BUN, and HA levels, and significant changes were observed for SCr and HA (Figure 7A).

HA-injected rats (5/6NX + HA group) exhibited increased tubulointerstitial fibrosis and glomerulosclerosis, compared with those in the 5/6NX group (Figure 7B). Consistent with these histological findings, the COL1A1-, VIM-, and ACTA2-positive areas were significantly larger in 5/6NX + HA rats, compared to 5/6NX rats (Figure 7B). These data indicate that HA promoted the progression of renal fibrosis in the rat model. Furthermore, SFN treatment (5/6NX + HA + SFN group) significantly reduced the fibrosis area, glomerular sclerosis index, and fibrotic protein levels (COL1A1, VIM, and ACTA2) compared with those in the 5/6NX + HA group (Figure 7B).

Overall, these data suggest that HA promotes renal fibrosis by disrupting the antioxidation function of Nrf2, consistent with the results obtained in HK-2 cells.

### 3.8. HA Induces Redox Imbalance by Disrupting the Antioxidation Function of Nrf2 In Vivo

**Figure 8:**
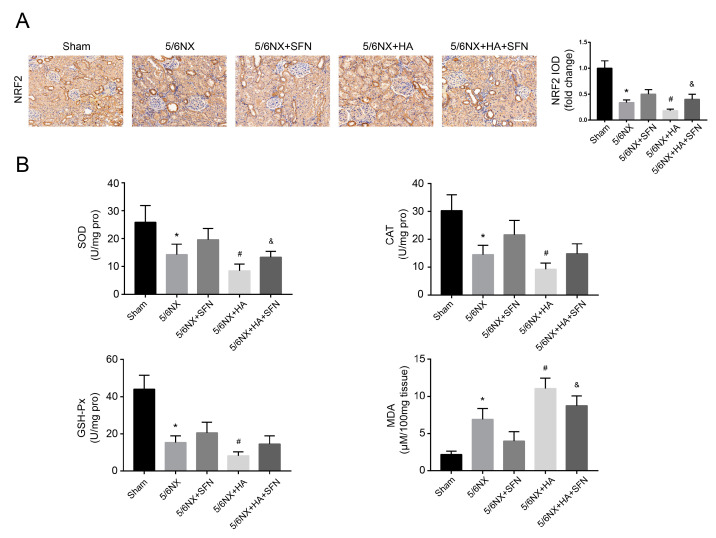
Effects of HA on redox imbalance in rat kidneys. (**A**) Immunohistochemistry for NRF2. Positive areas were quantified (right) by densitometry. Bar = 100 μm. (**B**) Levels of superoxide dismutase (SOD), catalase (CAT), glutathione peroxidase (GSH-Px), and malondialdehyde (MDA). Data are presented as the mean ± standard deviation (*n* = 9 per group). * *p* < 0.05 vs. sham group; # *p* < 0.05 vs. 5/6th nephrectomy (5/6NX) group; and & *p* < 0.05 vs. 5/6NX + HA group.

To investigate whether the redox imbalance induced by HA occurs in vivo, we determined the levels of MDA, NRF2, and antioxidant genes, including SOD, CAT, and GSH-Px. Immunohistochemical analysis revealed that 5/6NX + HA rats had much lower NRF2 levels compared to 5/6NX rats (Figure 8A). Moreover, the activities of SOD, CAT, and GSH-Px were decreased, whereas MDA levels were increased in 5/6NX + HA rats compared with those in 5/6NX controls (Figure 8B), suggesting that redox homeostasis was disrupted by HA. Furthermore, SFN treatment (5/6NX + HA + SFN group) attenuated the reductions in NRF2 and its downstream antioxidant genes levels, and elevation in MDA contents, compared to the 5/6NX+HA group, with significant differences observed for NRF2, SOD, and MDA (Figure 8A,B). These findings indicate that in the rat model, HA induced a redox imbalance by disrupting the antioxidation function of Nrf2, consistent with the results obtained in HK-2 cells.

## 4. Discussion

The nephrotoxicity of HA and its pathogenic mechanism have not been widely evaluated. Our results indicate that HA (1) promoted renal fibrosis progression; (2) activated ROS-mediated TGFβ/SMAD signaling; (3) caused a redox imbalance by impairing the antioxidant system; and (4) increased the degradation of ubiquitinated NRF2 by facilitating NRF2–KEAP1–CUL3 interactions, in HK-2 cells and 5/6NX rats. To our knowledge, this paper is the first to disclose a profibrotic role for HA, which acts by disrupting redox homeostasis.

In healthy kidneys, PBUTs are cleared through tubular secretion mediated by organic anion transporters (OATs) controlling the uptake of free PBUTs to tubular cells and excretion of PBUTs into the urine [35]. In patients with CKD, PBUTs accumulation exerts unique physicochemical properties and displays toxicity in the development of renal fibrosis. As important PBUTs, IS and PCS can promote the upregulation of fibrotic proteins and cytokines, epithelial mesenchymal-like transition, collagen accumulation, and renal fibrosis, in both nephrectomy animal model and HK-2 cells [17,36,37,38,39,40]. In this study, similar to IS and PCS, HA increased the levels of fibrotic proteins and induced an ECM imbalance in vitro, with similar results obtained in vivo. Moreover, we previously reported that *Eggerthella lenta*, an HA-producing bacterium, increased HA production and aggravated the progression of renal fibrosis in a 5/6NX rat model [41]. These findings provide compelling evidence that HA accumulation promotes the development of renal fibrosis in CKD.

A typical feature of renal fibrosis is high oxidative stress, a state involving uremic metabolite accumulation [42,43]. Previous studies reported that IS and PCS have strong prooxidant properties and can promote kidney injury and organ fibrosis by augmenting oxidative stress [17,44]. Another study reported that o-hydroxyhippuric acid induces ROS formation in renal tubular cells, inhibiting cell proliferation [45]. In this study, HA also showed prooxidant properties in cells and rats. Furthermore, the ROS elimination by antioxidants (NAC and DPI) significantly reversed HA-induced fibrotic marker expression in vitro, confirming that ROS play a direct role in HA-induced fibrotic responses. Numerous studies have reported that ROS, as intracellular second messengers, can promote organ and tissue fibrosis by activating redox-sensitive pathways, such as TGFβ/SMAD signaling [46,47], which is responsible for the initiation of fibrosis progression and activation of downstream fibrotic genes [48,49]. Here, antioxidants (NAC and DPI) significantly prevented HA-induced activation of TGFβ/SMAD signaling, suggesting that oxidative stress is the primary contributing factor to the HA-induced activation of TGFβ/SMAD signaling. Concurrent with our results, IS and PCS have been shown to activate TGFβ/SMAD signaling via increased oxidative stress [18]. Hence, our results indicate that HA promotes renal fibrosis in CKD by activating ROS-mediated TGFβ/SMAD signaling.

Oxidative stress occurs upon excessive ROS accumulation. The NRF2-driven antioxidant network can eliminate accumulated ROS, thereby preventing the initiation of oxidative stress [50]. Previous studies of PBUTs showed that the accumulation of IS, PCS, and indole-3-acetic acid is positively correlated with decreased NRF2 levels in patients with CKD [51]. Our results showed that HA suppressed the antioxidant network by downregulating NRF2 protein expression, indicating that HA attenuates ROS elimination and aggravates the redox imbalance. Concurrent with our results, another study reported that IS decreased the renal expression of NRF2 protein, leading to ROS accumulation [52]. Several studies have suggested that NRF2 is a critical target for renal failure. Cui et al. [53] and Shin et al. [54] emphasized that SFN attenuates cyclosporin A-induced nephrotoxicity and diabetic nephropathy by activating the antioxidative function of NRF2. Yoh et al. [55] demonstrated that knockdown of NRF2 aggravates oxidative stress and renal disease progression in diabetic mice. However, the association between PBUT-promoted renal fibrosis and loss of the NRF2-driven antioxidant system remains largely unclear. Through NRF2 gain- and loss-of-function experiments with SFN and NRF2-specific siRNA, respectively, we found that HA-induced fibrotic toxicity was inhibited by SFN and aggravated by NRF2 knockdown in vitro. Similarly, in vivo, SFN alleviated HA-promoted renal fibrosis. These consequences suggest that the dysfunction of the Nrf2 antioxidant defense network, caused by downregulation of NRF2, plays a significant role in HA-promoted renal fibrosis.

Currently, the mechanism underlying PBUT-mediated NRF2 downregulation remains unclear. Numerous studies have reported that the reduction in NRF2 levels is mediated by protein ubiquitination via the E3 ubiquitin ligase system [56,57]. In this system, KEAP1 is the substrate adaptor, CUL3 is the scaffold, and NRF2 is the substrate [58]. KEAP1 and CUL3 are negative regulators of NRF2 [58,59]. In this study, we found that HA accelerated NRF2 ubiquitination. However, KEAP1 and CUL3 expression levels were not significantly altered, suggesting that KEAP1 and CUL3 expression are not associated with HA-induced NRF2 ubiquitination. Two models of protein-protein interactions have been proposed to be responsible for E3 ubiquitin ligase activity [60]. The first is a hinge-latch model, suggesting that binding and dissociation between KEAP1 and NRF2 regulate the strength of ubiquitination, whereas the second model is a KEAP1-CUL3 dissociation model, suggesting that the association between KEAP1 and CUL3 regulates NRF2 ubiquitination. Herein, we found that HA strengthened the associations of KEAP1 with NRF2 and CUL3, indicating that HA increases E3 ligase activity by facilitating NRF2-KEAP1-CUL3 interactions. Concurrent with our results, Zhang et al. [61] reported that PAQRS strengthens NRF2 binding to KEAP1 to enhance E3 ubiquitin ligase activity, thus increasing the ubiquitination and degradation of NRF2. Villeneuve et al. [24] revealed that USP15 causes KEAP1 incorporation in the E3 ligase complex more efficiently, thereby increasing E3 ligase activity. Furthermore, enhanced protein–protein interactions in the E3 ligase complex potentially prevent NRF2 dissociation upon stimulation by ROS or electrophiles, ultimately preventing activation of the antioxidant system. Numerous studies have focused on how disturbing the interactions between Keap1 with Nrf2 or Cul3 result in the dissociation of NRF2 and inactivation of E3 ubiquitin ligase. However, our study revealed the opposite results, contributing to further improvements in relevant models. Overall, these findings indicate that HA increased E3 ligase activity by enhancing NRF2-KEAP1-CUL3 interactions, resulting in degradation of ubiquitinated NRF2.

Accumulating evidence indicates that KEAP1 is a direct site for small-molecule binding to the E3 ubiquitin ligase complex [59]. KEAP1 is a core component in the regulation of NRF2 ubiquitination and serves at least three functions by acting as a scaffold to anchor NRF2 in cytoplasm, as a sensor responding to oxidative/electrophilic stimulation, and as a substrate adaptor of E3 ubiquitin ligase to incorporate NRF2 into the KEAP1-CUL3 complex [61,62,63]. In this study, computer simulations indicated that HA directly docks into the active site of KEAP1 by interacting with the Tyr334 and Arg415 residues, which agrees with a previous report [64]. These results suggest that KEAP1 is a direct HA target responsible for HA-induced NRF2 ubiquitination. However, further studies are required to verify these results.

## 5. Conclusions

This study revealed that HA promotes the progression of renal fibrosis by disrupting redox homeostasis, which is maintained by the NRF2 antioxidant network. This negative impact is attributed to the ability of HA to induce NRF2 ubiquitination and degradation by enhancing NRF2-KEAP1-CUL3 interactions. These findings provide insight into the underlying mechanisms of PBUT-promoted renal fibrosis in CKD and suggest that the antioxidant system plays a key role in this mechanism. Furthermore, targeting the NRF2-driven antioxidant system may be an effective therapeutic strategy for preventing renal fibrosis progression and alleviating CKD deterioration.

## Supplementary Materials

The following are available online at https://www.mdpi.com/2076-3921/9/9/783/s1, Figure S1: Viability of HK-2 cells treated with 0–1000 μM hippuric acid (HA), Figure S2: Levels of reactive oxygen species (ROS) and hydrogen peroxide (H_2_O_2_) in HK-2 cells pretreated with 1000 µM *N*-acetylcysteine (NAC), 50 µM diphenylene iodonium (DPI), or the vehicle, followed by treatment with 1000 µM HA, Figure S3: Levels of ROS and H_2_O_2_ in HK-2 cells pretreated with 0–20 µM sulforaphane (SFN), followed by treatment with 1000 µM HA, Figure S4: Levels of ROS and H_2_O_2_ in HK-2 cells transfected with the control or *NRF2*-specific small interfering RNA (siRNA), followed by treatment with 1000 µM HA, Table S1: The primers utilized for a quantitative reverse transcription-polymerase chain reaction, Table S2: Small interfering RNA sequences applied in this experiment.
