# Supplementary material for: Hippuric Acid Promotes Renal Fibrosis by Disrupting Redox Homeostasis via Facilitation of NRF2–KEAP1–CUL3 Interactions in Chronic Kidney Disease
Source: Antioxidants (Basel). 2020 Aug 25;9(9):783. doi: 10.3390/antiox9090783 (PMC7555723; doi:10.3390/antiox9090783)
Supplement: Supplementary file 1 [file antioxidants-09-00783-s001.zip › antioxidants-891827-supplementary.docx]

**Supplementary Material**


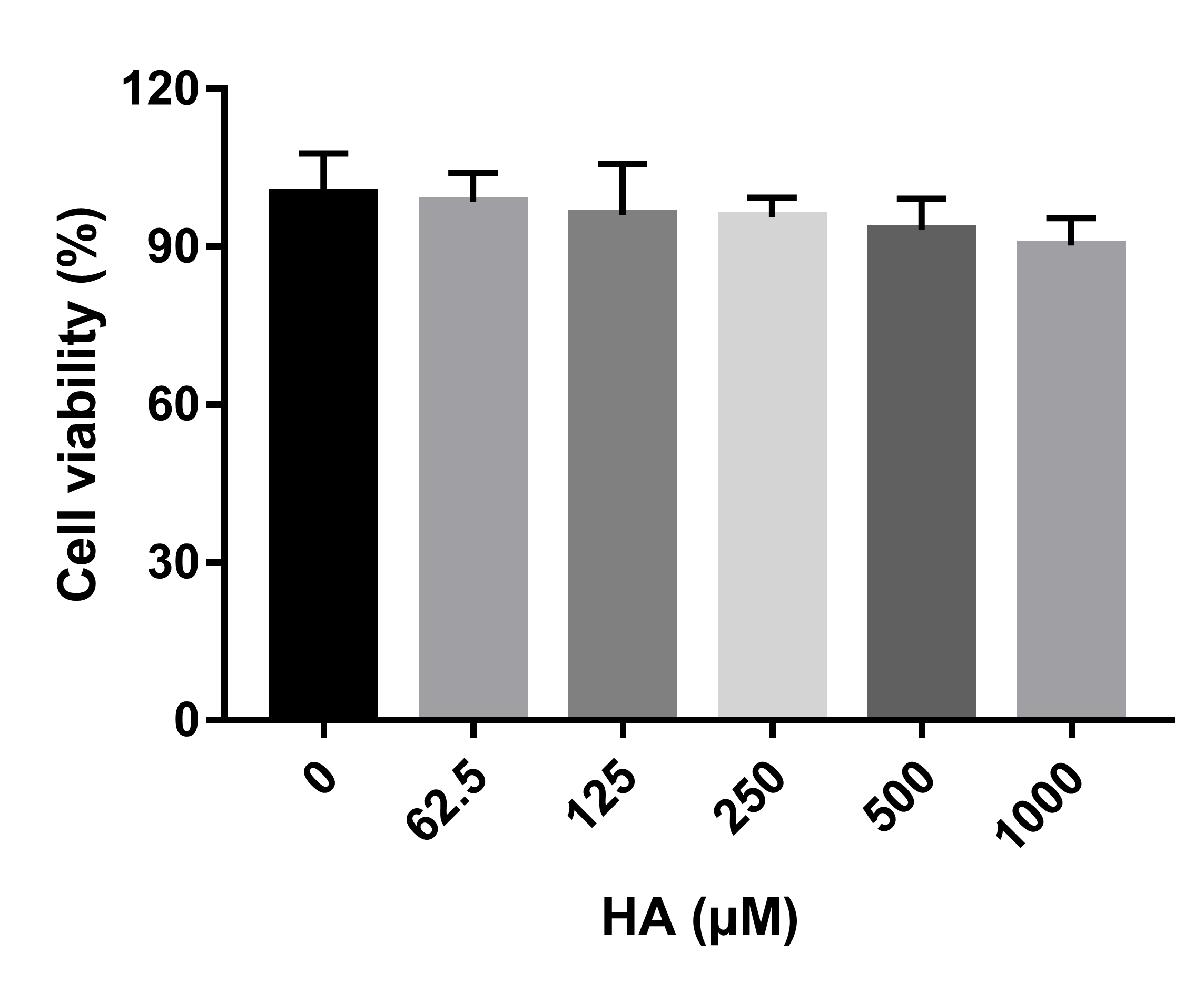


**Figure S1.** Viability of HK-2 cells treated with 0–1,000 μM hippuric acid (HA). Data are presented as the mean ± standard deviation.


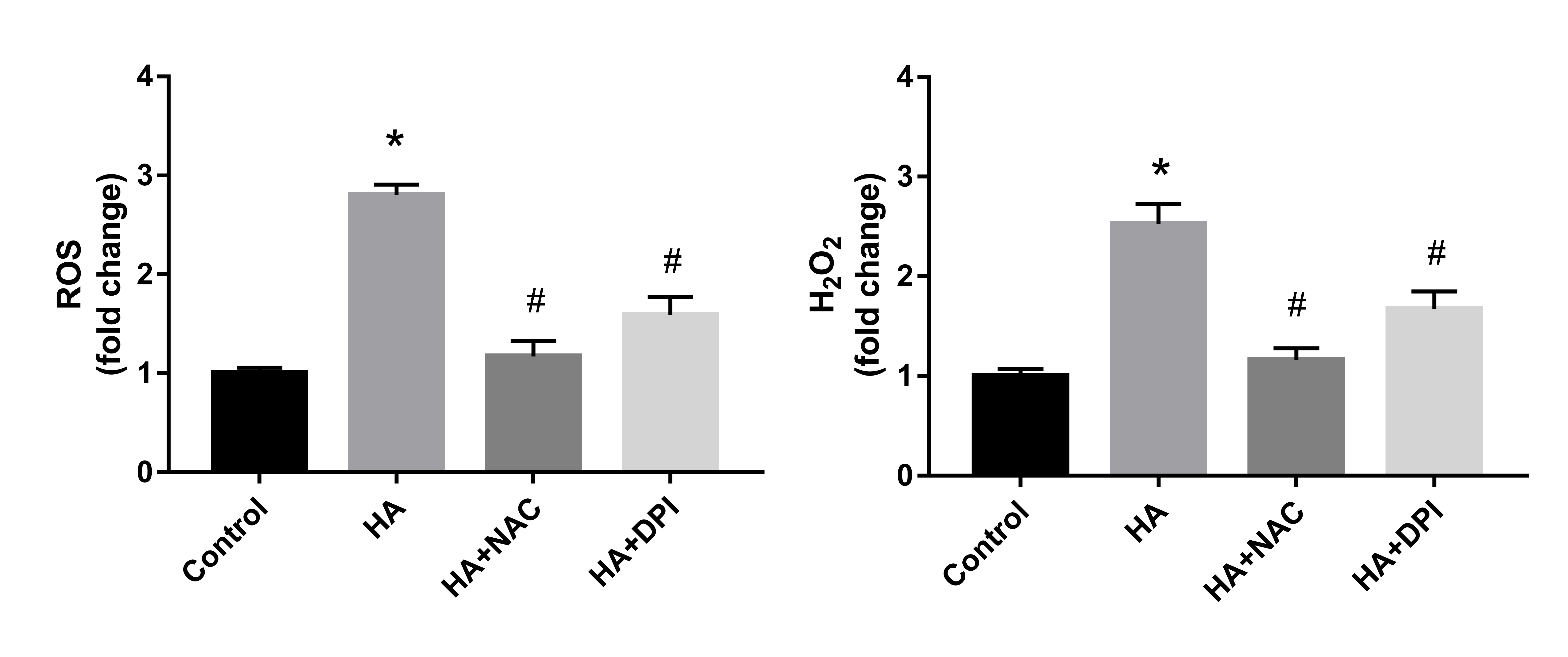


**Figure S2.** Levels of reactive oxygen species (ROS) and hydrogen peroxide (H_2_O_2_) in HK-2 cells pretreated with 1,000 µM *N*-acetylcysteine (NAC), 50 µM diphenylene iodonium (DPI), or the vehicle, followed by treatment with 1,000 µM hippuric acid (HA). Data are presented as the mean ± standard deviation. *P < 0.05 vs. control cells; #P < 0.05 vs. cells treated with HA only.


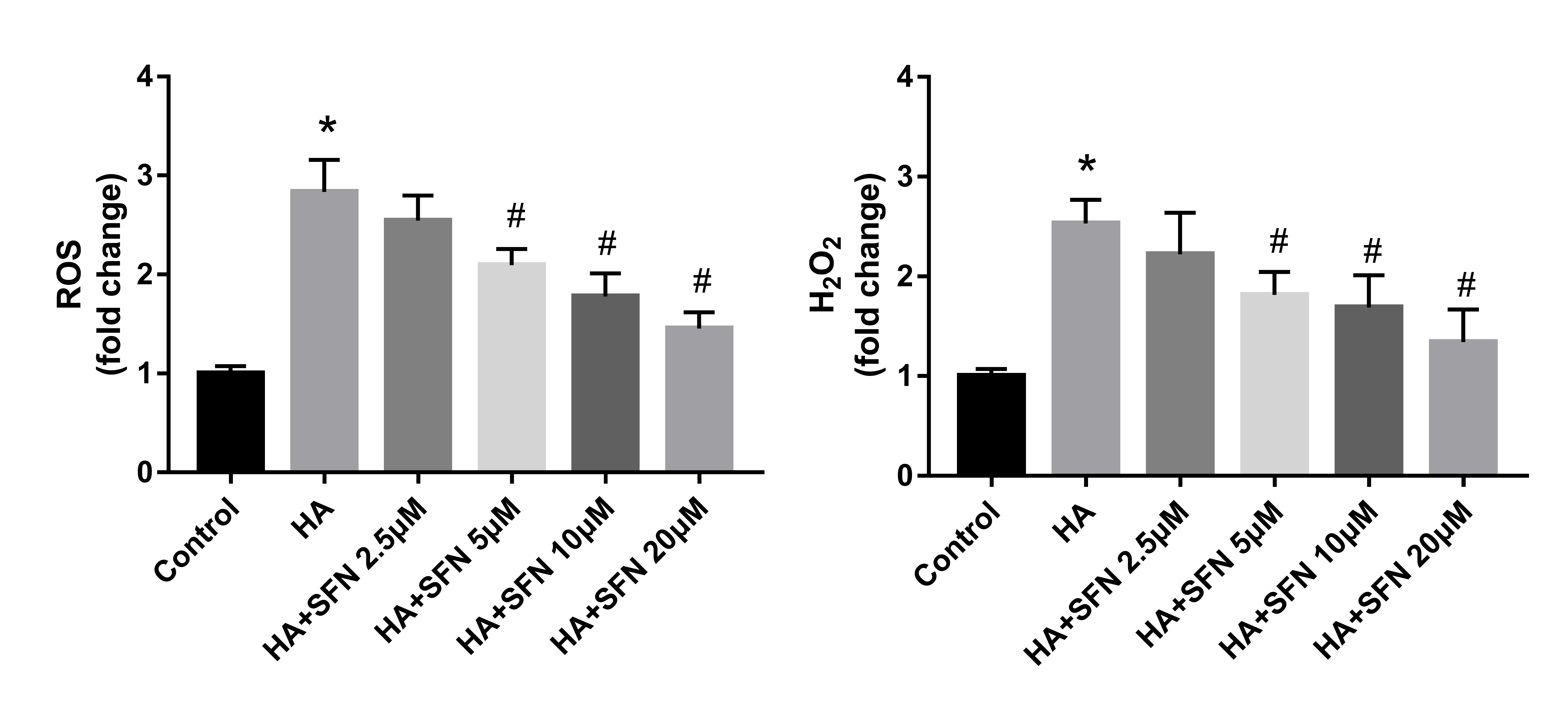


**Figure S3.** Levels of reactive oxygen species (ROS) and hydrogen peroxide (H_2_O_2_) in HK-2 cells pretreated with 0–20 µM sulforaphane (SFN), followed by treatment with 1,000 µM hippuric acid (HA). Data are presented as the mean ± standard deviation. *P < 0.05 vs. control group; #P < 0.05 vs. cells treated with HA only.


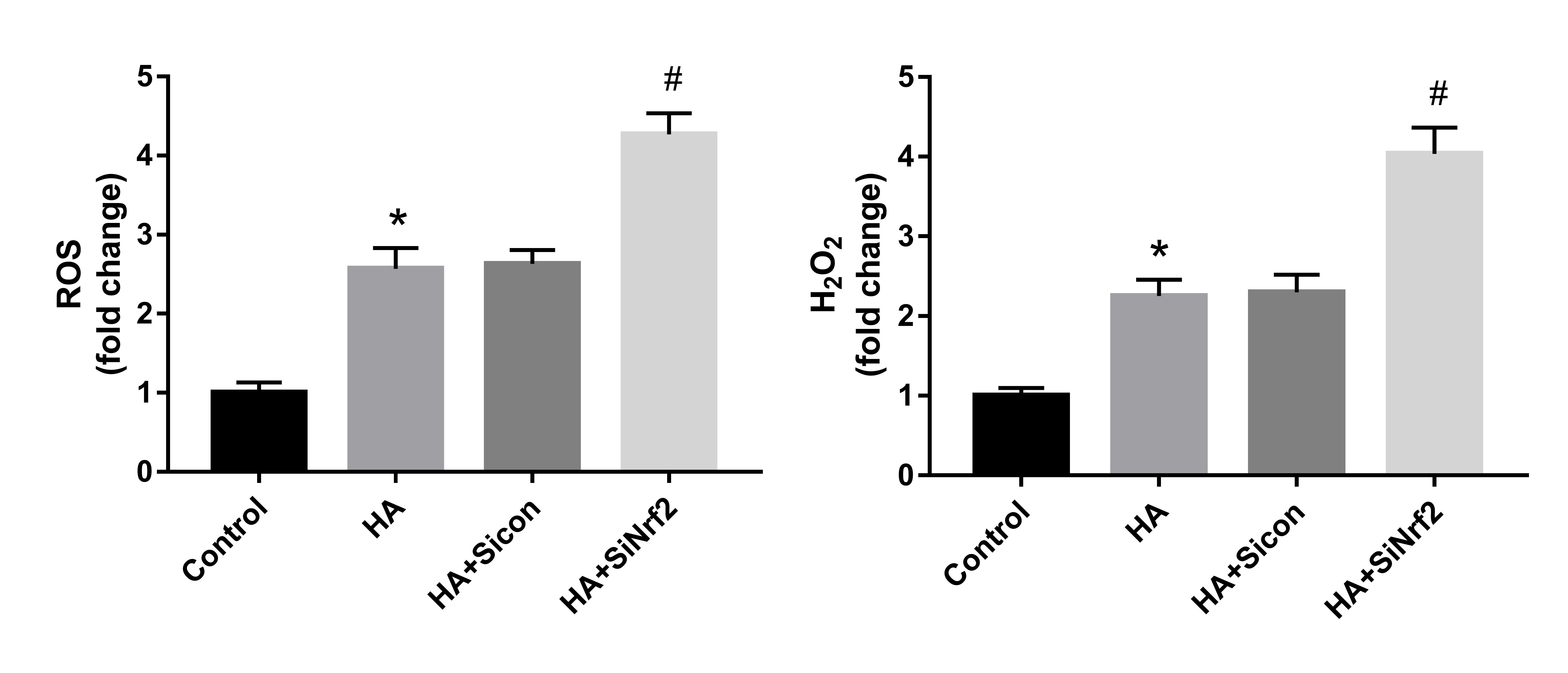


**Figure S4.** Levels of reactive oxygen species (ROS) and hydrogen peroxide (H_2_O_2_) in HK-2 cells transfected with the control or *NRF2*-specific small interfering RNA (siRNA), followed by treatment with 1,000 µM hippuric acid (HA). Data are presented as the mean ± standard deviation. *P < 0.05 vs. control group; #P < 0.05 vs. cells treated with HA + control siRNA.

**Table S1**. The primers utilized for quantitative reverse transcription-polymerase chain reaction.

| Human | Forward primer (5'→3') | Reverse primer (5'→3') |
| --- | --- | --- |
| *βACTIN* | TGGCACCCAGCACAATGAA | CTAAGTCATAGTCCGCCTAGAAGCA |
| *CDH1* | AGTCACTGACACCAACGATAAT | ATCGTTGTTCACTGGATTTGTG |
| *ACTA2* | CTCTGGACGCACAACTGGCATC | CACGCTCAGCAGTAGTAACGAAGG |
| *NOX4* | TCCAGTCCTTCCGTTGGTTTGCAG | TTGGGTCCACAACAGAAAACACCA |
| *COL1A1* | AAAGATGGACTCAACGGTCTC | CATCGTGAGCCTTCTCTTGAG |
| *VIM* | AGTCCACTGAGTACCGGAGAC | CATTTCACGCATCTGGCGTTC |
| *MMP9* | CAGTACCGAGAGAAAGCCTATT | CAGGATGTCATAGGTCACGTAG |
| *TIMP1* | CATCACTACCTGCAGTTTTGTG | TGGATAAACAGGGAAACACTGT |
| *NRF2* | TCCAAGTCCAGAAGCCAAACTGAC | GGAGAGGATGCTGCTGAAGGAATC |
| *HO1* | CCTCCCTGTACCACATCTATGT | GCTCTTCTGGGAAGTAGACAG |
| *NQO1* | GTCGGCAGAAGAGCACTGATCG | ACTCCACCACCTCCCATCCTTTC |
| *CUL3* | GACAAATCAACGGAAGAACCAA | TCCTTCTTCAGAAACAAGAGCT |
| *KEAP1* | ATTCAGCTGAGTGTTACTACCC | CAGCATAGATACAGTTGTGCAG |

**Table S2**. Small interfering RNA sequences applied in this experiment.

|  | Forward primer (5'→3') | Reverse primer (5'→3') |
| --- | --- | --- |
| Si*NRF2* | GGGAGGAGCUAUUAUCCAUTT | AUGGAUAAUAGCUCCUCCCTT |
| SiCon | UUCUCCGAACGUGUCACGUTT | ACGUGACACGUUCGGAGAATT |
